# The Financial Lives and Capabilities of Women Engaged in Sex Work: Can Paradoxical Autonomy Inform Intervention Strategies?

**DOI:** 10.5539/gjhs.v13n6p69

**Published:** 2021-05-08

**Authors:** Lyla S. Yang, Susan S. Witte, Carolina Vélez-Grau, Tara McCrimmon, Assel Terlikbayeva, Sholpan Primbetova, Gaukhar Mergenova, Nabila El-Bassel

**Affiliations:** 1 Columbia University, School of Social Work, New York, NY; 2 New York University, Silver School of Social Work, New York, NY; 3 Social Intervention Group, Columbia University School of Social Work, New York, NY; 4 Global Health Research Center of Central Asia, Almaty, Kazakhstan

**Keywords:** women engaged in sex work, FSW, drug use, financial lives, paradoxical autonomy

## Abstract

**Introduction:**

Despite growing attention to structural approaches to HIV prevention, including economic empowerment interventions for key populations, few studies examine the financial lives of women engaged in sex work (WESW) and even fewer examine the financial lives of those who also use drugs. The purpose of this paper is to examine the financial status, sex work involvement, and individual and structural vulnerabilities of women involved in sex work and drug use in Kazakhstan.

**Methods:**

We used baseline data from Project Nova, a cluster-randomized controlled trial that tested the efficacy of a combined HIV risk reduction and microfinance intervention for WESW in two cities in Kazakhstan. We collected data on income, savings, debt, sex work, drug use, homelessness, food insecurity, HIV status, attitudes towards safety, and financial knowledge from 400 participants through computer-assisted self-interview techniques. Descriptive statistics were utilized to describe and characterize the sample and aforementioned measures.

**Results:**

Findings illustrate the paradoxical nature of sex work, wherein women may achieve economic independence despite the great adversities they encounter in their daily lives and work. The majority of women (65%) in this study reported being the highest income earner in the household, caring for up to 3 dependents, and demonstrated entrepreneurial characteristics and aspirations for the future. However, many were still living below the poverty line (72.5%), as well as experiencing high levels of homelessness (58%) and food insecurity (89.5%).

**Conclusion:**

Study findings underscore the need for better understanding of the existing capabilities of WESW and those who use drugs, including financial autonomy and community supports, that may guide the design of programs that most effectively promote women’s economic well-being and ensure that it is not at the expense of wellness and safety. Designing such programs requires incorporating a social justice lens into social work and public health interventions, including HIV prevention, and attention to the human rights of the most marginalized and highest risk populations, including WESW and those who use drugs.

## 1. Introduction

In contrast to the decrease in new human immunodeficiency virus (HIV) infections globally, the epidemic continues to grow at a fast rate in Central Asia (UNAIDS, 2020). The majority of new HIV cases in Kazakhstan, the second largest Central Asian country by population, are diagnosed among specific populations, including women engaging in sex work (WESW) and people who inject drugs (The World Bank, 2019). A systematic review has estimated that WESW in low- and middle-income countries (LMIC) have more than 13 times greater odds of living with HIV than the general population of women (Baral et al., 2012). Among WESW, those who also use drugs experience heightened levels of risk that further increases their vulnerability to HIV (Baral et al., 2012; El-Bassel et al., 2014; Syvertsen et al., 2019). Economic vulnerabilities have been identified as a powerful motivator for women entering sex work, and while sex work can provide a platform for women to support themselves and their families, it is also a significant contributing factor in increasing women’s risk for HIV (Karamouzian et al., 2016; Reed et al., 2010; Scorgie et al., 2012). The importance of addressing the “upstream” drivers of HIV, such as poverty and gender inequity, has motivated a push towards structural HIV prevention interventions for WESW (Blankenship et al., 2006; Gupta et al., 2008; Shannon et al., 2018). Despite the calls for structural HIV preventions and the growing number of economic-strengthening interventions aimed toward WESW (Duvendack & Mader, 2020; Witte et al., 2015), there is only a limited number of studies that examine the financial affairs of WESW: that is, to what extent these women may already be achieving economic empowerment and how best to incorporate their existing occupational capabilities, skills, and experiences within transformative structural interventions to reduce their risk of HIV and other sexually transmitted infections (STI). Furthermore, to the best of our knowledge, no such studies exist among WESW who also use drugs.

Sex work engenders paradoxical autonomy for many women. The autonomy is paradoxical because to engage in sex work means exposure to a myriad of heightened risks, including violence, drug use, and STI and HIV, all in the effort to overcome economic hardship (Karamouzian et al., 2016; Shannon et al., 2015; Syvertsen et al., 2019; Tsai et al., 2013; Valdez et al., 2000). At the same time, WESW experience increased distance from traditional gender roles by sustaining employment and generating income through sex work, which in turn enables them to be the primary earner in their households (Cepeda, 2004; Valdez et al., 2000). This paradoxical autonomy framework has been applied in the service of understanding the dynamic lives of women who defy traditional gender roles (Cepeda, 2004; Nowotny et al., 2017; Valdez et al., 2000). Surveying the benefits of this increased autonomy, for example, a study among WESW in Mongolia found that most women were the primary income generator in their households and were able to financially support an average of three dependents (Tsai et al., 2013). WESW in Cote d’Ivoire earned income higher than the country’s estimated weekly per capita gross national income (Namey et al., 2018). A qualitative study among WESW in India identifies specific benefits that accrue to these women, including financial independence, flexibility, and control in enhancing women’s agency and empowerment through engagement with sex work (Swendeman et al., 2015). For some women in LMIC, sex work may be one of the only or best options available to them to provide basic needs for themselves and to prevent their families from falling further into poverty (Karamouzian et al., 2016; Nowotny et al., 2017; Tsai et al., 2013).

However, this autonomy is paradoxical because engagement in sex work does not fully relieve the women of multiple risks to their health and overall wellbeing (Nowotny et al., 2017; Syvertsen et al., 2019; Valdez et al., 2000). Studies have found that WESW experience high levels of gender-based violence, food insecurity, and homelessness. These risks and basic needs often lead directly to an increased risk of exposure to more proximal HIV-risk factors, such as multiple high-risk partners or condom use (Barreto et al., 2017; Footer et al., 2020). This is because WESW may experience limited bargaining power to negotiate safer sex practices, due to higher premiums offered for unprotected or riskier sexual acts (Lall et al., 2017; Sangram et al., 2016; Reed et al., 2010; Namey et al., 2018). Moreover, the intersection and interdependency between sex work and drug use exacerbate women’s vulnerability to HIV (Nowotny et al., 2017; Shannon et al., 2008; Shokoohi et al., 2019; Syvertsen et al., 2019). WESW who use drugs navigate their choices in unique risk environments and demonstrate considerable capacity for resisting circumstances shaped by economic and social conditions outside of their control (Cepeda, 2004; Valdez et al., 2000). Thus, structural interventions aimed at altering the economic contexts that have an impact on the high rates of HIV in this particular group of women who are actively engaged in the informal labor sector are necessary. Those who design such interventions can benefit from a clearer understanding of these women’s financial lives and contexts, so that their adaptation and implementation of interventions are more effectively tailored to the lived experiences of WESW.

To address this gap between a full understanding of WESW’s lives and effective implementation of interventions, this study explores the financial lives and capabilities of women who both trade sex and use drugs in Kazakhstan. In this paper, we employ the concept of paradoxical autonomy to organize our analysis and further our understanding of the complex financial lives of these women who operate and survive within challenging socioeconomic contexts. The questions guiding this descriptive study include: (1) How do the women engage in sex work and manage their finances? (2) What micro and macro levels of vulnerabilities do they face? (3) How do they perceive financial security? To move away from the deficit discourse of WESW and drug use, we apply an empowerment lens, which allows us to highlight how women utilize their social resources and exercise agency. We emphasize women’s empowerment as a crucial factor in informing structural HIV prevention programming in order to more effectively respond to these women’s needs and capabilities (Kabeer, 1999).

## 2. Methods

### 2.1 Study Design

This cross sectional study draws on the baseline data from a larger trial called Project Nova. Project Nova is one of the first randomized controlled trials to test a combination of HIV-prevention and economic strengthening intervention for WESW who also use drugs. Recruitment and baseline data collection were conducted between 2015 and 2018. Eligible women were those reporting that they were over 18 years old; had engaged in illicit drug use within the past 12 months; had provided sex in return for money, goods, drugs, or services within the past 90 days; and had at least one incidence of unprotected sex within the past 90 days. Women were not eligible for the study if they were unable to communicate in Russian, planning to relocate away from the study site within the next year, or were unable to provide informed consent due to cognitive impairment. Of 763 women assessed for eligibility 401 (57%) completed the baseline assessment and 400 were included in this study (due to missing data for one). For a more detailed description of the parent study protocols, intervention, and main outcomes, please see El-Bassel et al. (in press), McCrimmon et al. (2018), and Mergenova et al. (2019).

### 2.2 Sampling

Recruitment took place in two cities in Kazakhstan: Almaty and Temirtau. A description of the geographical and population differences between these sites is available elsewhere (McCrimmon et al., 2018; Mergenova et al., 2019). We recruited participants via field recruitment visits and a peer-referral program utilizing network-based recruitment. Research assistants approached potential participants by conducting regular visits to non-governmental organizations, HIV clinics, drug treatment clinics, saunas, hotels, and street-based sex work venues. They were provided with a brief overview of the intervention and were asked for verbal consent to conduct a screening for study eligibility. To extend recruitment further into communities of WESW, eligible participants were offered a small financial incentive (US$5) to refer their friends and acquaintances to be screened for the study through a system of paper coupons. Eligible participants were scheduled for study intake procedures, including informed consent and the baseline assessment at the field office locations. The Institutional Review Board at Columbia University and the Ethics Committee at the Kazakhstan School of Public Health approved all study protocols, instruments, and materials, and one community advisory board at each study site was engaged to provide input and feedback on study protocols.

### 2.3 Data Collection

Baseline assessments were administered in field office locations by research assistants. Computer-assisted self-interviewing was used to conduct behavioral questionnaires and a clinical coordinator collected biospecimens for HIV and STI testing. Participants received the equivalent of US$10 for participating in both interview and biological testing procedures (McCrimmon et al., 2018). Within 2 weeks of completing the baseline assessment, participants were enrolled in groups of six to eight each, then randomized and followed as a cohort over time (McCrimmon et al., 2018). This paper utilizes results from the behavioral questionnaire that describe demographic and other variables associated with sex work, risk behaviors, and the financial context of participants’ lives.

### 2.4 Measures

#### 2.4.1 Socio-Demographic Characteristics; Individual and Structural Vulnerabilities

Baseline socio-demographics variables reported here include the women’s age, ethnicity, level of education, whether or not the women have a main partner or children, marital status, and housing status. Recent experience of homelessness was measured by asking if the participant had been homeless or without a regular place to sleep in the past 90 days. Recent experience of food insecurity was measured by asking if the participant had not had enough money to buy food in the past 90 days. The women were asked about their recent HIV test results, with options to report whether they tested positive or negative, the test was indeterminate, they did not get back results, or they preferred not to say. The women were also asked about whether they used drugs in the past 90 days and whether they had ever experienced drug overdose. The Alcohol Use Disorders Identification Test (AUDIT) was used to measure alcohol use, with a summed score of 8 or more indicating harmful alcohol consumption (Saunders, Aasland, Babor, DeLaFuente, & Grant, 1993). The AUDIT scale demonstrated good internal consistency (Cronbach’s alpha = .907).

#### 2.4.2 Work, Household Earnings, Financial Dependents, and Sex Work

Work status was assessed using multiple choice items, including: full-time, part-time, seasonal, government assistance, and other. Household earnings were assessed by asking who in the household currently earns money, who currently makes the most money, and the average amount of total monthly household income, as well as average monthly income earned solely by the participant. Income was reported in local currency, Kazakhstan Tenge (KZT), and converted to US dollars using the average rate during the period of data collection of 363.21 KZT to 1 dollar (MBH Media, Inc., 2018). The number of financial dependents was measured by asking how many people depended upon the participant for the majority of their food and/or shelter. A new variable estimating income per person was created by dividing the participant’s average monthly income by the number of people who depend on her financially, including herself. This variable was recoded to determine the number of women living below or above the poverty line (21 825 KZT) as specified by the Kazakhstan Ministry of National Economy.

Several questions asked about women’s engagement in sex work, including number of years in business, age of entry into sex work, and what strategies they used to solicit clients. Additionally, we asked whether women have a boss, have co-workers, and keep all their earnings from sex work. A follow-up question inquired about with whom women share their sex work income. Women were also asked whether they have been offered more money for sex without a condom and the frequency of this request.

#### 2.4.3 Savings and Debt

Women were asked whether they currently have any savings and, if so, the amount and its location (e.g., formal bank or at home). A new variable representing women’s savings as a percentage of their income was created by dividing the amount in participants’ current savings by their average monthly income. Women were asked whether they currently owe any money. If women answered yes, follow-up questions were asked: the amount currently owed, to whom they owe money, and reasons for borrowing money.

#### 2.4.4 Financial Security and Future Safety

Financial security and financial self-efficacy were assessed by items from the Domestic Violence Related Financial Issues Scale (Weaver et al., 2009). Participants rated the level of impact financial security, employment, or educational opportunities have on their current and future safety using a 5-point scale, with possible answers ranging from 1 (very strongly agree) to 5 (very strongly disagree). Additionally, women were asked to rate their confidence level in meeting their goals for becoming financially secure using a 5-point scale, with possible answers ranging from 1 (extremely confident) to 5 (not at all confident). The financial security and future safety scale had a high level of internal consistency with an alpha of .89.

#### 2.4.5 Financial Knowledge

The financial knowledge scale consists of 19 items. High scores represent higher knowledge. This scale was adapted from the Financial Education Core Curriculum (Microfinance Opportunities, 2002). Cronbach’s alpha showed these items to reach acceptable reliability, with an alpha of .897.

### 2.5 Data Analysis

Data was analyzed using SPSS (version 26). Descriptive statistics were utilized to provide simple descriptive summaries of the sample and the aforementioned measures.

## 3. Results

### 3.1 Socio-Demographic Characteristics; Individual and Structural Vulnerabilities

Table 1 reports the socio-demographic characteristics of study participants. The participants’ ages ranged from 18 to 61 years, with a mean age of 34.12 years (SD = 8.4). The majority of the participants were Russian (67.3%). Most respondents had completed high school or above (68.2%). At the time of the interview, 31% of participants reported being single or never married, and over half had a main partner (52.3%). Nearly two-thirds reported having children (62.5%).

Over half of the participants either owned or rented the home or apartment in which they lived (56.0%). Few women (4.5%) reported living in an institution or on the street; however, recent experiences of homelessness and food insecurity were high: 58.0% reported that they have been homeless and 89.5% had not had enough money to buy food in the past 90 days. Sixty-nine women out of 381 reported HIV-positive status from a recent HIV test. The majority of participants reported recent drug use and over a third (34.0%) reported injection drugs in the past 90 days. One-hundred and fifty women reported experiencing drug overdose. Out of the 392 participants who completed the AUDIT, 69.4% scored higher than 8, indicating harmful alcohol consumption. Out of the 265 participants who have been offered more money for condomless sex, 54.0% reported that it happened during half or more than half of their paid sexual encounters.

### 3.2 Work, Income, and History of Engagement in Sex Work

Women were most often the highest income earners in their household, and over three quarters of women reported one or more adults or children who financially rely on them. Participants endorsed multiple work status options but most (72.6%) worked part time or seasonally, while few (5.8%) received government assistance. Women reported other sources of income and support, including having a male sponsor, stealing, begging, and receiving help from various family members. On average, participants earned 46 801.50 KZT (approximately 125.49 USD) in a month, while average household monthly income is 63 743.75 KZT (170.92 USD). According to the Kazakhstan Ministry of National Economy, the poverty line in Kazakhstan is 21 835 KZT per person. By these estimates, most participants and their households (n=290, 72.5%) were living below the poverty line.

Most women indicated sex work as their primary source of income. The mean number of years participants had been engaged in sex work is 10 years (SD = 7.4); duration ranged from 0 to 40 years. Approximately half (49.5%) had been working in sex work for 9 years or less and 27.2% had been engaging in sex work for over 15 years. The average age of entry into sex work was 20.8 years (SD = 5.5, range 12–48). One hundred and eighty women (45.0%) were 18 or younger when they first exchanged sex for money, goods, drugs, or other services. Almost ninety percent of the women operated their sex work business without a boss who managed them. The women found clients mostly by using the phone (63.7%), on the street (29.5%), and through acquaintances (27.7%). The vast majority of the participants reported keeping all their earnings from sex work. However, 73 women reported sharing their sex work income with a partner (21.9%), boss (20.5%), or friend (19.2%).

### 3.3 Savings and Debt

Only eight participants reported having any savings. Among the eight participants who saved, half of them held more than 100 000 KZT (269 USD) in savings and the other half had less than 20 000 KZT (54 USD). Five women saved more than 100% of their average monthly income. In contrast, the majority of participants (83%) currently owed money with almost half (47.8%) reporting that they owed more than 100% of their monthly income. Women reported borrowing money from a number of different places and people. The most common ways were to borrow from friends (29.3%), formal institutions (21.5%), family members (16.0%), moneylenders (10.8%), store credit (8.0%), and co-workers (7.5%). In Almaty, forty-nine women (23.0%) out of 213 reported borrowing money from a neighbor. Women accrued debt for a variety of reasons, although the majority used it to pay day-to-day living expenses (57.5%).

### 3.4 Financial Security and Future Safety

Participants strongly endorsed perceptions that improving their financial situation might influence their current and future safety. Most women (93.3%) agreed that becoming financially secure would increase their safety and 92.8% agreed that increasing their ability to save would increase their safety. An even higher percentage agreed that adequate employment (95.6%) and access to education opportunities (95.6%) would increase their current and future safety. However, 59.7% expressed being somewhat to extremely confident in meeting their goals for becoming financially secure, while 40.3% were not at all or not very confident.

### 3.5 Financial Knowledge

Overall, the participants in our sample reported having fair to good knowledge about finances. On a 19-item financial knowledge scale with a theoretical range of 0 to 18, in which higher scores reflect greater financial knowledge, the median score was 13. The preponderance of women (72.6 %) scored between the ranges of 8 to 17 and 3.2% answered all the questions correctly.

## 4. Discussion

Findings from this study, using the baseline data from a larger intervention trial (Mergenova et al., 2019), illuminate the financial lives and working environments of WESW and those who use drugs in Kazakhstan. Research on WESW and drug use often focuses on their vulnerabilities and risk factors, yet our findings support previous suggestions that the paradoxical nature of sex work allows the women to demonstrate economic independence and agency despite the great adversities they encounter in their lives and work. The majority of the women in this study reported being the highest income earner in the household, with an average of 3 people, children and adults, who depend on them for financial support. The average tenure in sex work of 10 years suggests that women may be making deliberate choices to stay in sex work. Alternatively, they may be persistently left without options to maintain income while decreasing their own interpersonal risks for HIV infection, substance and/or alcohol dependence, and intimate and community violence. Swendeman et al. (2015) note in their study among WESW in India that although women initially may have been coerced into sex work, they report exercising personal agency in choosing to continue in order to gain greater financial autonomy, flexibility, and control over their bodies.

Furthermore, the women also demonstrated highly entrepreneurial characteristics and aspirations for the future. Nine out of ten women operated their sex work business without a boss, pimp, or madam who managed them, while a majority of women also kept all the money they earned from sex work. Nearly all women aspired to become financially secure and obtain adequate employment and education to improve their current and future safety. However, women’s confidence in meeting their goals was low, indicating a critical need for intervention: increasing self-efficacy to promote transfer of existing skills meaningfully into new forms of income generation, and identifying resources to support this transition. These findings are consistent with studies that provide more nuanced narratives of WESW and advocate for women’s agency in choosing sex work. WESW in various contexts refer to themselves as businesswomen, request training in entrepreneurship and business development, and take pride in their capabilities to fulfill multiple intersecting identities, including those of mother and provider (Basnyat, 2020; Ding, 2020; Leddy et al., 2020; Swendeman et al., 2015).

Our findings also support the importance of community and familial support among WESW. The women in the study demonstrated tremendous resilience as they navigated informal systems of support by borrowing money from family, friends, and neighbors to make ends meet, and/or bartering resources that allowed them to care for dependents while earning income. Being able to receive instrumental support from the community was particularly critical to the women as they reported having very little savings, large sums of debt, and distrust towards financial institutions (Vélez-Grau et al., 2020). Additionally, many women reported working alongside other WESW, providing a sense of community. These findings suggest that WESW have existing informal systems of support that can be capitalized upon to enhance interventions aimed at reducing financial vulnerability. A study in India among 2,400 WESW found that women who believed in their collective power to bring about positive change and fundamental rights are less likely to report financial vulnerability and more likely to save for future emergencies (Patel et al., 2016). Moreover, studies among WESW have also illustrated the positive impact of collective agency on stigma associated with sex work, suggesting that by mobilizing together through shared experiences, women feel less isolated and more empowered to make choices to their benefit, such as negotiating condom use and managing finances (Bavel, 2017; Mantsios et al., 2018; Scorgie et al., 2012).

While the resiliency of the women in this study and their capacity to provide for themselves and their families should be highlighted and celebrated, it should also be viewed as an active and dynamic resistance to social and economic barriers that limit their choices in life (Kabeer, 1999; Park et al., 2020). In the qualitative component of this study, women’s narratives reflected that they would not engage in sex work if they had alternative opportunities and options. However, these women are still choosing sex work despite the punishingly high cost, which includes detrimental health risks and social stigmatization due to their social position that limits their opportunities (Basnyat, 2020; Karamouzian et al., 2016; Vélez-Grau et al., 2020). Social stigma may be a major barrier to women’s access to other opportunities. Similar to numerous studies that examine the vulnerabilities of WESW who use drugs, we also found high rates of homelessness, food insecurity, and income below the poverty line (Shokoohi et al., 2018, 2019; Syvertsen et al., 2019). While the majority of women and their households are estimated to be living below the poverty line, only a few reported receiving any government assistance. A report from the United Nations Children’s Fund (UNICEF) of Kazakhstan revealed that multiple barriers exist for low-income households to obtain poverty-targeted social assistance, such as lack of awareness of these families’ need, restrictive eligibility rules, and onerous documentation requirements (UNICEF, 2017). Further research should incorporate intervention components that can loop into existing systems to assist WESW with gaining access to governmental support.

Our findings underscore the importance of offering economic empowerment or support programs in conjunction with HIV risk reduction options so that women must not choose between survival and safety. These findings point to a number of microfinance intervention components that may alleviate some of the challenges to seeking alternative employment for women, such as the ability to accumulate savings and pay off debt, the opportunity to learn new vocational or business development strategies, and the need to integrate resource and referral mechanisms to increase women’s access to local or national government assistance. These are the foci of some current combination-intervention strategies, including Project Nova. The question is whether this will be enough.

Despite women’s capacities to survive within the unfair environments that put them at the intersection of marginality, we should not expect them to thrive; we should be actively trying to change these social/political/economic environments (Park et al., 2020). The ability to make meaningful choices between alternate desirable options is required for women’s empowerment (Kabeer, 1999). Thus, economic empowerment for HIV prevention among WESW must not only attempt to incorporate components that address structural-level issues which, when alleviated, can enhance resources available to women and thus heighten their agency and achievements. These interventions must also incorporate WESW’s own lived experiences into building the most effective responses, while researchers take a more critical position with regard to empirical approaches for study. This could be achieved, for example, by expanding from health- or asset-based theories to human rights-based approaches. This shift in emphasis aims to elevate community empowerment by facilitating meaningful involvement of women in the process of program development and enhancing collective agency among women who work alongside one another (Leddy et al., (2020).

## 5. Limitations

There are limitations that need to be considered. First, the quantitative nature of this study may not be as effective in capturing a nuanced understanding of women’s lived experiences and the intricacies of their financial status. Having access to qualitative data, such as financial diaries, to supplement our baseline data would have allowed us to further explore how drug use may impact women’s financial decisions and the different ways women engage in sex work together. Second, the convenience sampling method was used in two cities in Kazakhstan to recruit participants, which limits generalization of the findings to a broader population living at the intersection of sex work and drug use. Further research from other settings is recommended to expand on the resiliency and entrepreneurial themes that have emerged from this study. This would provide more strength-based narratives of WESW who are operating within challenging socioeconomic contexts.

## 6. Conclusion

This study elucidated the paradoxical nature of the financial lives of WESW and drug use. The findings underscore the need for better understanding of the existing capabilities and community support of WESW that may guide the design of programs that not only most effectively promote women’s economic well-being, but also ensure sustainability through the process of collective empowerment. Moreover, HIV prevention interventions must move beyond consideration of the public health aspects to incorporate a social justice lens to ensure that we are advocating for the human rights of WESW.

## Figures and Tables

**Table 1.** Socio-demographic characteristics; individual and structural vulnerabilities (N=400)

Variable	Percentage (n)
Age (mean, range)	34.12 (18–61)
Ethnicity	
Russian	67.3% (269)
Other (Kazakh, Tatar, Ukrainian, and others)	32.7% (131)
Level of education	
Less than high school	31.8% (127)
High school/vocational, university, or beyond	68.2% (273)
Currently have a main partner	52.3% (209)
Marital status	
Single or never married	31.0% (124)
Married or common law marriage	26.5% (106)
Divorced/separated/widowed	42.5% (170)
Have children under the age of 18	62.5 (250)
Housing	
Own home	20.5% (82)
Rent	34.5% (138)
Live in parent’s, family member’s, or someone else’s home	40.5% (162)
Live in institution/street/other	4.5% (18)
HIV Positive (n=381)	17.3% (69)
Drug use in the past 90 days	86.0% (344)
Injection drug use in the past 90 days	34.0% (136)
Ever overdosed on any drugs	37.5% (150)
Harmful alcohol use with AUDIT score > 8 (n=392)	69.4% (272)
Homeless in the past 90 days	58.0% (232)
Food insecure in the past 90 days	89.5% (358)
Offered more money for sex without a condom half or more than half of the time (n=265)	54.0% (143)

**Table 2.** Work, income, history of engagement in sex work (N=400)

Variable	Percentage (N)
Work Status (Includes endorsement of multiple items)	
Full Time	9.3% (37)
Part Time Or Seasonal	72.6% (290)
Government Assistance	5.8% (23)
Highest Household Income Earner (Check all that apply)	
Respondent	65.0% (260)
Husband, Boyfriend, Or Other Primary Partners	11.8% (47)
Another Adult And Other	26.8% (107)
Total Number Of Child & Non-Child Dependents (Mean, SD)	3.1 (2.5)
Total Monthly Household Income (Mean, SD)	63 743.75 KZT (57 265.09) 170.92 USD (153.55)
Total Monthly Participant Income (Mean, SD)	46 801.50 KZT (47 542.16) 125.49 USD (127.48)
Total Monthly Participant Income From Sex Work (Mean, SD)	36 691.52 KZT (42 178.15) 98.38 USD (113.09)
Estimated Income Per Head	
≤ 21 835 KZT (Below Poverty Line Around 59 USD)	72.5% (290)
Sex Work As Participant’S Main Source Of Income	63.3% (253)
Keep All Money From Sex Work (N=393)	81.4% (320)
Age Of Entry Into Sex Work (Mean, SD)	20.8 (5.5)
Number Of Years In Sex Work (Mean, SD)	10.0 (7.4) (Range 0–40)
Client Contact And Solicitation Strategies (Check all that apply)	
Found Through Phone	63.7% (255)
Found On Street	29.5% (118)
Found Through Acquaintances	27.3 (109)
Found Through Internet	17.8% (71)
Have A Boss/Pimp/Madam Who Manages Participant’s Sex Work	10.3% (41)
Work Alongside Others Who Engage In Sex Work	42.0% (168)

**Table 3.** Participant Savings and Debt (N=400)

Variable	Percentage (n)
Participant currently has any savings	2.0% (8)
Participant currently owes anyone money	83.0% (332)
Percentage of participant’s income currently owed	
0%	20.0% (80)
< 99%	32.2% (129)
≥ 100%	47.8% (191)
To whom participants owe money (Top 3)	
Friends	29.3% (117)
Formal institution	21.5% (86)
Family member	16.0% (64)
Reasons for borrowing money (Check all that apply)	
Day-to-day living	57.5% (230)
Family events	18.3% (73)
Paying off own debt	17.3% (69)
Emergency (Non-health related)	12.5% (50)
Other (Healthcare, educational or employment related)	29.2% (116)

**Table 4.** Financial security and future safety

Variable	Total sample (n=400) Percentage (n)
Becoming financially secure would be helpful in increasing my current and future safety*	
Agree	93.2% (373)
Increasing my ability to save would be helpful in increasing my current and future safety*	
Agree	92.7% (371)
Adequate employment would be helpful in increasing my current and future safety*	
Agree	95.5% (382)
Access to educational opportunities would be helpful in increasing my current and future safety*	
Agree	95.5% (382)
How confident are you that you can meet your goals for becoming financially secure?	
Somewhat, very, and extremely confident	59.7% (239)
Not very and not at all confident	40.3% (161)

*Note*. Answers were combined into two options: agree (somewhat, strongly, and very strongly agree) and disagree (strongly and very strongly disagree).
